# Percussion Tenderness as a Sign of Phthisis

**Published:** 1910-03-12

**Authors:** 


### PERCUSSION TENDERNESS AS A SIGN OF PHTHISIS.

Montgomery has made an extensive study of tenderness on percussion in diseases of the chest, including pulmonary tuberculosis, at the Henry Phipps Institute. In France the subject was mentioned by Chomel, Broussais, Andral, Louis and Laennec; in England by Graves, Stokes, John Forbes, and somewhat later by Charles West, Walshe, and Wilson Fox. In Germany, perhaps the earliest reference to tenderness on percussion was made by Ziemssen. Among both earlier and later writers tenderness is regarded by some as a valuable symptom, by others as of little value, whilst by some it is entirely ignored.

Various methods have been employed to elicit tenderness, such as light touch, percussion, pressure, rubbing, and various manipulations of the tissues, one method sometimes evoking tenderness when other methods fail. The tenderness noted in patients at the Phipps Institute was obtained by percussion, and was inquired into during or after the routine percussion for the percussion note, so that the examination was not materially prolonged. Elaborate tables are given which refer to 100 cases of uncomplicated pulmonary tuberculosis, mostly moderately and far advanced. Some were arrested, some incipient, some apparently cured. Only one examination of each case is recorded. Tenderness occurred in nearly half of the cases, and was present a little oftener on the left than on the right side. The extent of the tenderness on the left side was considerably greater than on the right side. Tenderness was present oftener anteriorly than posteriorly, oftener on the left side anteriorly than on the right, oftener in the combined axillary and infra-axillary region on the left side than on the right. Posteriorly the tenderness was about equally distributed on the two sides, and more extensive lesions were found on the left side than the right at the autopsies. Out of the 100 cases, 21 showed tenderness at the apices in front above the clavicles, and 18 at the apices behind in the suprascapular regions—a large proportion of the total 47 cases with tenderness. The tenderness was found more often above the left clavicle than above the right, and more often in the right suprascapular region than in the left.

At autopsies the left apical pleura was anteriorly involved more frequently than the right, and the right apical pleura was posteriorly involved more frequently than the left.

### What Tenderness Means.

Tenderness at the apices, as at other parts of the chest, may occur over an acute lesion, but sometimes marked circumscribed tenderness is present when the process has been a long time healed, and after positive evidences of a lesion have disappeared. In cases with general chest tenderness there may be more marked tenderness over a tuberculous lesion. One patient with a lesion at the right apex later became so nervous and restless that the condition could not be demonstrated. There was general bodily tenderness, but it was much more marked at the right apex. Circumscribed tenderness at the apices can usually be attributed to tuberculosis when no other cause can be found, especially when the tenderness persists.

Tenderness elsewhere than at the apex may occur when the physical signs of a lesion are not distinct, or before the signs become marked. The left mammary region was tender 18 times and the right only eight times, probably because the pleura in the left mammary region becomes earlier involved in the majority of cases. The presence of the heart on the left side may at times explain the tenderness on this side.

Tenderness is comparatively infrequent over the lower portions of the chest posteriorly, in spite of the thinness of the chest wall here; also over the right infra-axillary region, and over the lower right chest anteriorly, probably because of the relative infrequence of involvement of these localities in these cases, and at the autopsies the lower parts of the lower lobes are the parts least involved.

### Special Cases.

A few cases of special interest deserve mention. A far advanced case with gangrene of the lung had very marked tenderness over the upper portion of the right chest anteriorly and in the axilla. Autopsy revealed gangrene of the lung with destructive involvement of the tissues of the chest wall beneath the area of tenderness.

In cases of tuberculous empyema Montgomery observed little or no tenderness with the possible exception of one case that was never positively diagnosed as tuberculous, in which there was marked localised tenderness over a small area between two ribs where the fluid pointed. In cases of pneumothorax tenderness is usually present at the time of onset, and may be so marked that even the lightest percussion is unbearable.

In two cases of pericarditis, supposed to be secondary to inflammation of the pleura, no special aid was obtained from the tenderness. One of the cases showed very marked tenderness all over the front of the chest, while the other revealed no thoracic tenderness except over a small area in the second left interspace over a cavity. A case which showed both pleuræ thickened and the pleural cavities completely obliterated at autopsy, only occasionally had tenderness over the lower portion of the left chest in front, or in the infra-axillary region, though it was looked for on several occasions during some months before death. The lungs were extensively diseased.

